# Barriers and facilitators to implementing a multilevel, multicomponent intervention promoting colorectal cancer screening in health centers: a qualitative study of key informant perspectives

**DOI:** 10.1186/s12913-024-10749-y

**Published:** 2024-03-29

**Authors:** V. M. Telles, S. Rodriguez, M. Torres, J. Schneider, J. Haughton, M. Maldonado, E. Arredondo

**Affiliations:** 1 Joint Doctoral Program in Public Health at San Diego State University, University of California, San Diego, USA; 2 Department of Anthropology, University of California, Riverside, Riverside, USA; 3 School of Public Health, San Diego State University Research Foundation, San Diego, USA; 4 Department of Family and Social Medicine, Albert Einstein College of Medicine, Bronx, USA; 5 Psychology Department, San Diego State University, San Diego, USA

**Keywords:** Colorectal cancer screening, Group-based intervention, Implementation science, Health equity, Multilevel, Multicomponent (ML-MC), Multicomponent (ML-MC)x

## Abstract

**Background:**

Colorectal cancer (CRC) continues to be a major cause of death in the U.S. despite the availability of effective screening tools. U.S. Latinos have lower rates of CRC screening and higher rates of death due to colorectal disease compared to non-Hispanic whites. Federally Qualified Health Centers (FQHCs) serve medically underserved populations, including many Latino patients. Given the low CRC screening rates, identifying culturally sensitive and cost-effective methods of promoting screening is a priority for many FQHCs.

**Methods:**

We interviewed FQHC leaders and providers using the Consolidated Framework for Implementation Research (CFIR) to identify barriers and facilitators to implementation of a multilevel, multicomponent (ML-MC) CRC screening intervention (i.e., *promotor* navigation and group-based education) in FQHCs. A rapid qualitative analysis approach was used to identify themes organized according to the following CFIR constructs: intervention characteristics, outer and inner settings, and characteristics of the individual.

**Results:**

We completed interviews with 13 healthcare professionals in leadership positions at six FQHCs. The participating FQHCs perceived the ML-MC screening CRC program as feasible and expressed interest in implementing the program at their sites. Facilitators included financial incentives for increasing screening rates, the need for patient education programming, and involving *promotores* to support the work of clinical teams. Barriers included concerns about available resources to implement new programs, lack of federal reimbursement for health education, competing priorities of other health concerns, and the need for more resources for confirmatory screening and treatment following a positive screen.

**Conclusions:**

FQHCs provide essential primary care to millions of underserved patients in the U.S. and have the ability and motivation to provide screenings for colorectal cancer. Partnering with an academic institution to deliver a group-based, *promotor*-led CRC screening intervention for patients not up to date with screening could help increase screening rates. By identifying the specific barriers and facilitators to implementing CRC intervention, findings suggest that group-based, promotor-led interventions are a promising approach.

**Supplementary Information:**

The online version contains supplementary material available at 10.1186/s12913-024-10749-y.

## Background

Colorectal cancer (CRC) is highly curable if detected early, but in the U.S. screening rates remain low, particularly among medically underserved populations [1]. CRC is 90% curable with timely detection and appropriate treatment of precancerous growths [2]. If not found until a patient is symptomatic, however, survival rates drop to 50% [3]. Epidemiologic data show disproportionate rates of CRC screening among Hispanic/Latinos, [4–6] who frequently receive their medical care at federally qualified health centers (FQHCs) [4, 6, 7]. In 2021, FQHCs served over 30 million patients across the U.S. of whom 20% were uninsured, 63% were racial/ethnic minorities, and 90% were living below 200% of the federal poverty level [8]. FQHCs and other safety-net healthcare systems face numerous challenges to achieving the National Colorectal Cancer Roundtable goal of screening at least 80% of adults for CRC in U.S. communities [9, 10]. 

The Guide to Community Preventive Services [11] recommends several evidence-based strategies to increase CRC screening. When considering patient-level factors, the Guide recommends one-on-one education, reducing structural barriers, and small media (i.e., educational pamphlets). Although the Guide notes insufficient evidence to support group education to promote CRC screening [11], emerging research suggests this may be a promising approach in underserved communities [12]. Group education has the potential to reach communities at highest risk for CRC as it can help address psychosocial barriers (e.g., fear of finding cancer), distrust of the medical system, and enhance facilitators of CRC screening (e.g., social support, group cohesion). In addition, group education provides an opportunity to reach a larger number of individuals and decrease costs due to the efficiency of modality. This approach utilizes peer and social support to navigate the complex CRC screening process and address patient barriers such as fear of test results [5, 13–20]. 

At the provider level, the Community Guide recommends increasing access to fecal immunochemical tests (FITs) through outreach, including provider trainings/feedback and reminders for due or overdue screening [11]. At the system level, the Community Guide recommends strategies, such as mailed FITs and electronic health records, prompts to remind providers that a patient is due for his/her screening, and ways to streamline the ordering of CRC screening [11]. Recent systematic reviews comparing the effectiveness of patient, provider, and system-level strategies on CRC screening outcomes [21, 22] found that mailed FIT programs utilizing multilevel, multicomponent (ML-MC) interventions (e.g., mailed FIT and education) are effective in improving CRC screening disparities among low-income, racially diverse populations. ML-MC interventions are community-engaged interventions that operate on multiple levels simultaneously and involve multiple intervention components that are synchronized across levels [23]. Emerging research suggests that multilevel approaches to increasing CRC screening can improve screening rates for Latino communities. A study combining community health worker (CHW) -led patient navigation and group education among Latinos in FQHCs has shown increases in CRC screening compared to those in the control group [24]. To achieve the CRC screening goal of 80%, the Community Guide recommends ML-MC strategies.

Juntos Contra el Cáncer/Together Against Cancer (JUNTOS), a promotor-led, group-based intervention to increase CRC screening among Latinos, successfully completed screening of 66.7% of its participants by 6 to 9 months post-intervention [12]. JUNTOS utilized multiple components (i.e., *promotores*, group-based education) to target various levels of influence (i.e., individual, interpersonal, organizational, community) that affect behaviors related to CRC screening among Latinos. Results from this study suggest that JUNTOS was feasible to implement in community and health center settings and increased the uptake of CRC screening among Latinos. To expand the reach of ML-MC programming for CRC screening, we sought to understand perceptions of barriers and facilitators to implementing ML-MC interventions (i.e., *promotor* navigation and group-based education) among additional FQHCs in San Diego County.

Informed by the Consolidated Framework for Implementation Research (CFIR) [25], the current study aims to describe barriers and facilitators, as well as provide recommendations for implementing a ML-MC intervention in FQHCs based on interpretation of the findings. We examined the following CFIR domains: intervention characteristics, outer and inner settings, and characteristics of the individual. In addition, we evaluated the organizational-level characteristics (e.g., readiness, capacity) that may facilitate or interfere with implementing a ML-MC CRC screening program.

## Methods

### Setting

Individual (*n* = 3) and group-based (*n* = 3) interviews occurred in several FQHCs in San Diego County, California. San Diego County has over 3 million residents, 35% of whom are Latino persons, 37% speak a non-English language, 23% are foreign-born, and 11% live in poverty [26]. All interviews were conducted at each FQHC’s administrative office, except for one conducted via telephone when an in-person meeting was not feasible. The study was approved by the San Diego State University Institutional Review Board.

### Guiding framework

We used the original CFIR by Damschroder et al. to develop questions that assessed constructs that were hypothesized to influence intervention implementation and effectiveness [25]. CFIR comprises 27 theory-informed constructs arranged within 5 domains: intervention characteristics, outer setting, inner setting, characteristics of individuals, and process [25]. Only CFIR constructs relevant to the implementation process of a ML-MC CRC screening intervention were included to inform the development of the interview guide (see Table 1).

**Table 1:** CFIR and non-CFIR domains assessed in the interview guide

CFIR Domains	Description^a^
**Intervention characteristics**	Key attributes of the intervention influencing the success of implementation (e.g., cost, cultural sensitivity)
**Characteristics of the individual**	Individuals’ beliefs, knowledge, attitudes about, and value placed on the intervention and personal attributes of implementors that may affect implementation.
**Inner setting**	
*Available resources*	The level of resources dedicated to implementation and on-going operations (e.g., money, training, space, CRC screening processes)
*Organizational incentives & rewards*	Extrinsic incentives (e.g., goal-sharing awards, promotions) and less tangible incentives (e.g., respect)
*Relative priority*	Individuals’ shared perception of the importance of implementing a CRC screening program compared to other initiatives within the FQHC
*Culture of the health center*	Norms and values regarding CRC within the FQHC
*Goals & Feedback*	The degree to which internal goals for CRC screening are clearly communicated, acted upon, and fed back to staff
*Networks & Communication*	The nature and quality of social networks and formal and informal communications within a FQHC
**Outer Setting**	
*Cosmopolitanism*	The degree to which an FQHC is networked with other external organizations or academic institutions
*Patient Needs & Resources*	The extent to which patient needs, as well as barriers and facilitators to meet those needs, are accurately known and prioritized by the FQHC
*External Policy & Incentives*	External strategies to spread interventions, including policy and regulations (governmental or other central entity), external mandates, recommendations and guidelines, pay-for-performance, collaboratives, and public or benchmark reporting

^a^ Descriptions taken from Damschroder et al. 2009 and tailored for the study

### Recruitment

FQHCs located in San Diego County were eligible to participate in the study. FQHCs were ineligible to participate if they were already involved in CRC screening research or programming during the recruitment phase. Research assistants contacted key informants (K.I.) at 15 FQHCs via email and phone to schedule a one-hour interview. Of those FQHCs that responded, nine were excluded because they were unable to participate during the timeframe or did not meet the eligibility criteria. The final sample consisted of six FQHCs.

### Data collection and analysis

Between late July and early October 2019, we interviewed K.I.s at each FQHC. K.I.s were given a one-page overview of the components of the ML-MC program before the meeting and a copy of the consent form. The overview outlined the key components and processes involved in the program (bilingual didactic workshops delivered by *promotores*, target demographics, patient navigation for CRC screening). K.I.s reviewed materials before the interview to gain a basic understanding of the proposed ML-MC CRC screening intervention, which encompassed both group-based education and a promotor-led navigation component. This pre-interview preparation allowed for the opportunity to ask any questions regarding the ML-MC and equipped K.I.s with the knowledge necessary to respond to questions regarding potential implementation of the intervention at their FQHC.

During each one-hour interview, we used a semi-structured interview guide (developed for this study) that included topics related to patient demographics, current health prevention programs, current or prior research partnerships, CRC screening efforts, CRC screening processes across all phases of patient care, CRC treatment plans for the uninsured, and CRC screening rates, goals, or milestones (See Additional File 2). Our guide also included questions assessing barriers and facilitators according to CFIR domains. Each interview included one to four K.I.s from an FQHC, depending on the availability and interest of staff.

Two members of the research team conducted the interviews. All interviews were audio recorded and five research assistants transcribed them verbatim and spot-checked for accuracy. We used rapid assessment methods [27, 28] to summarize and synthesize all interviews. We developed a summary template with headings based on the questions in the interview guide, using CFIR domains (see Table 1) and non-CFIR constructs (e.g., patient demographics and other observations) were included to provide contextual information about the FQHC. Non-CFIR constructs were not included as codes during analysis. Relevant CFIR constructs and subconstructs were identified as a priori codes. Four graduate student researchers independently summarized each interview and compared their summaries in groups of four and five, discussed disagreements, and reached a consensus before moving forward [27]. We transferred the summaries from all interviews into a matrix sorted by domain to compare and contrast interviews and begin topic monitoring for analysis. E.A., J.H., M.T., and S.R. reviewed the matrix to identify four major emergent themes guided by CFIR domains: characteristics of individuals; outer setting; inner setting; and characteristics of the intervention (see Table 1).

## Results

This study included 13 healthcare professionals in leadership positions at six FQHCs. K.I.s in leadership positions encompassed Medical Directors, Quality Improvement Directors, Research Directors, staff directly involved in coordinating care for patients such as registered nurses and physicians, and other administrative roles at some facilities such as managerial positions in business development, research, or grant efforts. Table 2 presents the number of key informants interviewed, as well as demographic characteristics of the FQHCs interviewed taken from Health Resources & Services Administration’s (HRSA) Uniform Data System (UDS) [29]. The participating FQHCs varied in size, serving between 9,000 and 167,900 patients in the San Diego County region in 2021 [29]. While all the participating FQHCs reported having some type of CRC screening protocols and processes, none reported having any education-based programming to promote CRC screening to their patients. Results are organized according to CFIR domains, which encompass barriers and facilitators to the implementation of a ML-MC CRC screening program in FQHCs. Additional File 1 summarizes the barriers and facilitators to implementing a ML-ML CRC screening program for each of the CFIR domains, along with key quotes. Quotes that we illustrative and representative of the key facilitators/barriers reported by K.I.s were chosen by the team to include in the table.

**Table 2:** Demographic Characteristics of FQHCs (2018)

FQHC	# of Health centers	Key informants (*N*)	Size of Population Serving	UDS-Operating Budget	CRC Screening Rate	% of Latinx Pts	% of Patients Uninsured
01	8	1	25,000–50,000	22 million	44.36%	50.74%	13.83%
02	8	4	50,001-100,000	58 million	38.39%	61.18%	31.03%
03	23	1	100,000 +	182 million	42.16%	59.79%	29.26%
04	2	1	Under 25,000	8.6 million	53.39%	66.91%	8.72%
05	13	3	50,001-100,000	71 million	53.68%	63.56%	21.83%
06	23	4	100,000 +	220 million	26.56%	63.99%	5.08%

### Intervention characteristics

Within the intervention characteristics domain, cost relating to the intervention and implementation was the main code identified. While K.I.s were interested in implementing the program in their respective health centers, funding for and reimbursement was identified as an implementation facilitator. One K.I. described the long-term reimbursement benefits of intervention implementation by noting how involving participants more in their care through programs like this will engage them to continue seeking care. The fact that the program is CHW-led, which is reimbursable, was also appealing to FQHCs.

### Characteristics of individuals

Within the characteristics of individuals’ domain, motivations and buy-in from individuals, as well as knowledge and beliefs about the intervention, were identified as a code. Most FQHCs reported enthusiasm and interest in implementing a ML-MC CRC screening program. K.I.s expressed that such a program could help bridge the gaps in health education and would be a welcomed added resource for patients. One K.I. noted the potential for a program like this to challenge cultural norms around the importance of CRC screening, noting how placing emphasis on a particular issue can help create and institutionalize a culture of its importance. A few K.I.s also expressed the value they placed on implementing a pre-established program due to the challenges associated with designing an intervention from within the health center. Taken together, the findings highlight that this program aligns with the health center’s goals.

### Inner setting

The main codes identified within the inner setting domain were available resources, organizational incentives and rewards, relative priority, culture, goals and feedback, and networks and communication.

#### Available resources

This code describes resources specific to a ML-MC CRC screening program’s implementation and ongoing operations (e.g., money, training, space, CRC screening processes).

All participating FQHCs reported having existing or limited health education initiatives; however, the majority of programs focused on diabetes prevention and intervention. Of those FQHCs that reported having limited health education programs, two expressed the desire and need to expand current infrastructure for health education, sharing that patients request classes or group education opportunities. None of the K.I.s reported any cancer screening health education currently offered in their respective health centers.

In general, most health centers reported having the capacity and a certain level of resources available to dedicate to a ML-MC CRC screening program including, human resources/staff available, training, physical space, and CRC screening processes and follow-up. However, a few health centers reported barriers such as, limited funding and space, limited staff time and turnover, competing priorities, and lack of leadership infrastructure as potential barriers to implementation readiness.

K.I.s described past and present resources that serve as predictors or indicators for patient access to CRC screening within the context of implementing a ML-MC program. These resources included: outreach/”in-reach,” internal teams focused on patient care metrics, and free or discounted FIT kits and colonoscopies. Several FQHCs described the following outreach processes to increase CRC screening: (1) patient outreach/”in-reach” efforts that included targeted or mass blind mailing of FIT cards, (2) texting/calling patients, and (3) campaigns for those who need or are due for screening. “In-reach” was described as CRC screening efforts that take place once the patient arrives at the health center. These processes were often prompted by alerts from electronic medical records (EMRs) that inform providers or care coordination teams daily about gaps in care for patients between the ages of 50 and 75 who have not had a colonoscopy in the past 10 years or a FIT test in the past year. One K.I. described an internal quality improvement team at their health center that focused on specific patient care and metrics and an outreach team that communicated their efforts at local events. Additionally, the same health center offered free FIT kits for patients who paid cash. Another health center used a health center-based grant to fund colonoscopies. One health center offered a robust stipend program for colonoscopies where almost all the fees were paid for, and patient paid $100, and they had an agreement with an organization that covers 10 free colonoscopies donated to the health center in a year. For uninsured or underinsured patients, this would alleviate high deductible payments. Colonoscopies are usually covered by a patient’s health insurance, with patients paying for a copayment and/or deductible based on their plan (i.e., Medicaid). The same health center also provided discounted FIT kits and patient navigators to assist their population with the CRC screening processes.

Existing human resources such as, staff and *promotors* were identified as a facilitator for some health centers. Most FQHCs reported that the most operative CRC screening resource involved medical assistants (M.A.s) as patient navigators for CRC screening and follow-up. M.A.s often function as the primary source of education and information for CRC screening; however, instructional materials for patients are often not available in other languages and health centers are looking to expand resources in alternate languages: for example, one K.I. described M.A.s giving screening test materials and guidance to participants in Spanish, then sending them home with written instructions in English only. Another health center reported having *promotor* capacity to educate patients about CRC screening that could facilitate implementation of the program; they described previous training that their promotores had received about cancer screening and noted the desire to expand these efforts and improve how well their impact is tracked.

A few health centers used their awareness of capacity and space limitations for program implementation to inform a solutions-oriented approach. One health center reported that they usually cap health education workshops at 20–25 participants and noted that barriers to implementation could be reduced by planning ahead and to ensure available space and resources. In addition to space limitations, barriers relating to limited staff/personnel dedicated to implementing a ML-MC program, as well as high turnover were reported. Health centers reported limited time for staff to implement existing CRC screening procedures and the high turnover could result in delayed projects and difficulty implementing a new program. Half of the health centers expressed a desire for more robust health education components. One health center noted that they do not have the teams to structure and execute a ML-MC CRC screening program. The capacity for health education programming was low in one health center due to limited leadership positions to sustain the programs. For example, one FQHC had a group medical visit pilot program that was stopped due to challenges with leadership infrastructure (i.e., department chairs and directors). Additionally, limitations in EMR systems, such as the limited capacity to increase internal processes related to CRC screening, can result in frustrations for program implementation. In contrast, one health center had a new EMR system that they noted would provide them greater visibility of patient “gaps.” Another health center had a region they served with minimal access to a gastroenterologist specialist. There was only one gastroenterologist in the region, creating various barriers for patients, such as limited appointments for colonoscopies, high costs for uninsured patients, and transportation difficulties. Knowing this, they increased their efforts in annual CRC screening.

Lastly, funding for educational programs was a challenge reported across the majority of health centers. Several K.I.s expressed interest in implementing a ML-MC program but were restricted due to a lack of funding and staff time. One health center stated that often they need more funding to move forward with program planning and find funding later to implement projects. As such, the inclusion of *promotores* or patient navigators provided by the external program implementation team was received positively by some health centers, while others reported having their own group of *promotores* and coordinators who receive training on CRC screening and hospital workflow. Given the limited capacity of M.A.s, shifting the responsibility of preventive care education to *promotores* would allow the health center and administration to operate more efficiently.

##### Organizational incentives and rewards

 K.I.s mentioned specific extrinsic incentives such as goal-sharing awards, bonuses for providers, performance reviews, and promotions. One health center has a robust reporting system broken down into care teams that are widely shared, distributed, and transparent in the organization. Care teams that are performing well are rewarded thus prompting other care teams to engage in competition to improve their performance. Another health center discussed meeting certain metrics that are tied to each health center administrator’s performance and evaluation annually, which reward providers with bonuses if there is improvement in CRC screening rates. Some health centers also remind providers of meeting Healthcare Effectiveness Data and Information Set (HEDIS) [30, 31] and other quality measures as part of their assessment but offer no incentive. Several health centers had the capacity for patient and staff incentives and one shared that they have seen CRC screening increase considerably with the use of participant incentives. Other forms of incentives to increase the patient return rate of screening tests were raffles for a gift (e.g., tablets) or friendly competition between staff members. No barriers related to this code were reported.

##### Relative priority

Prioritization of ML-MC CRC screening interventions over other initiatives differed among each FQHC. One health center noted that they value one-on-one interactions over group education; however, three health centers noted that group education was a top priority for their 2020 work plans. Another health center revealed that their health education programs typically target patients already diagnosed with a condition, such as those living with diabetes or other chronic conditions. Rather than providing CRC screening education, this health center included health messaging around the importance of screening in waiting rooms. This sentiment was echoed by K.I.s from other health centers that reported a higher priority to educate those already diagnosed with chronic conditions than focusing on preventative care. Majority of health centers reported competing priorities from external policies such as meeting metrics for other diseases; however, one health center stated that they can figure out how to make anything work if it is seen as valuable and necessary.

##### Culture of the health center

The health center’s culture includes the norms, values, and assumptions of the implementing FQHC. Approximately half of the health centers were receptive to a ML-MC CRC screening program and shared their internal environment’s values and norms. Two health centers discussed the importance of quality and integrity in their respective health centers, specifically reporting on the value placed on partnering with community organizations and research institutions to focus on population health programs. One K.I. described the importance of shaping the health center culture to “institutionalize” the importance of prevention among both staff and patient. Another health center described the importance of their quality of care based on their mission of supporting an underserved, disenfranchised community. Still, because only one participant was present, it was difficult to differentiate whether it was a collective value or an individual one. Surprisingly, that same health center was hesitant to support prevention-focused programs. Two health centers did not have responses about their organizations’ norms or values.

##### Goals and feedback

 A few K.I.s described how their FQHCs communicated their goals for increasing CRC screening rates and provided feedback to staff on progress towards goals. For example, one health center used data from quality measure assessments to improve screening rates while another had an internal goal for increasing CRC screening rates that involved exceeding federal/state goals (HEDIS and UDS). All the FQHCs with internal goals aimed to at least hit the 50% federal/state screening rates, if not higher. One FQHC wanted to qualitatively follow up on its internal goals by capturing stories of the impact that early screening has had on individuals with positive cases. Lastly, one K.I. described their efforts to convene a multidisciplinary, internal task force where they brainstormed ways to increase screening, communicate feedback, and provide incentives to staff.

##### Networks and communication

While a few health centers reported the importance of having provider and care team huddles for patient preparation and navigation in their workflow routines, only one had a department dedicated to increasing patient engagement and retention through phone outreach to diverse populations. One health center discussed the need for *promotores* to integrate screening conversations into their workflow and build relationships with staff and providers to increase patient efficiency and continuity of care.

### Outer setting

The codes identified within the outer setting domain were cosmopolitanism, patient needs and resources, and external policy and incentives.

#### Cosmopolitanism

Cosmopolitanism captures how an FQHC is networked or connected with outside organizations. Health center partnerships varied, but FQHCs reported being a member of a larger entity such as Integrated Health Partners (IHP), working with school districts, churches, health plans or homeless centers, and various university-based initiatives. One FQHC emphasized the importance of and desire to build external relationships with social service agencies to address social determinants of health and diverse patient populations. Another FQHC reported that they had fewer external networks by comparing their partnerships to neighboring FQHCs elucidated by using phrases such as “ahead of us” and “playing catch up.” Moreover, several K.I.s noted the value of maintaining good relationships with specialty health centers to provide low-cost service for uninsured patients. However, another K.I. reported that the time required to build and maintain relations is a barrier.

#### Patient needs and resources

Prioritizing patient needs and resources to access CRC medical services was noted by health center staff. For example, only providing free FIT kits to patients did not address transportation, time, and adherence to screening (health literacy) barriers. A few health centers also expressed the difficulty of engaging patients in preventative care efforts including time as a barrier to attending an appointment, let alone a workshop. One K.I. described previous workshops offered and emphasized how difficult it was to get patients to attend and follow up for screening completion. Two health centers stated that there is a patient desire for more group-based education and value placed on in-person connection and building support and community around these topics, especially among Latino communities. The responses about patient needs helped gauge the level of FQHC commitment to their patients’ needs and the likelihood of them acting on it, especially for CRC screening efforts.

#### External policy and incentives

External policies and procedures can positively or negatively impact an organization’s collective evaluation of their task demands, resource availability, and other situational factors, such as the timing of the CRC-group-based intervention (Weiner et al., 2009).

The FQHCs reported that mandates from federal agencies like HEDIS or Uniform Data Systems (UDS) [29] measures are significant motivators for implementing CRC screening programs. One K.I. discussed the important financial implications of meeting HEDIS measures, noting the impact of this metric on provider and administrator performance evaluations and describing the opportunity to receive bonuses for improved rates. Another K.I. reported their ability to fund screening initiatives through funding provided through health plans to reach HEDIS measures.

Several K.I.s also noted incentives from IHP. Insurance companies incentivize CRC screening efforts or other preventative measures such as diabetes, obesity, and behavioral health. In some cases, IHP was able to offset costs (i.e., buying FIT cards and sending them out for testing) and aggregate rates to determine if there is a need for outreach for that population based on how each health center is performing. Another external policy is funding/reimbursement for group sessions from insurance companies. Group sessions are only reimbursable if the patient is seen by a provider (CHWs not included). Having a reimbursement component similar to one-on-one education would allow the possibility of CRC group-based education programs to be sustainable after grant funding ends. Lastly, societal values were recognized as an important dimension of health systems and health system change; one health center noted how difficult it is to fund preventive care without grant funds because of the *“fee for service”* system.

## Discussion

This study identified barriers and facilitators to implementing a promotor-led, group-based, ML-MC CRC screening program in FQHCs in San Diego County, CA, through interviews with FQHC leaders, providers, and staff. Our findings show that most K.I.s were enthusiastic about implementing a group-based promotor-led program in their respective health centers and reported having the capacity to do so. FQHCs provide essential primary care to underserved racial/ethnic communities [32] with historically low cancer screening rates due to multilevel barriers to accessing quality care [33]. Given their strong relationships in their communities, FQHCs are uniquely positioned to implement promotor-led programs and provide culturally- and linguistically-appropriate CRC education. Findings from this study can be used to inform collaborative partnerships between academic institutions and FQHCs to develop strategies targeting barriers and facilitators to implementation. Table 3 summarizes recommendations for implementing a ML-MC CRC screening program in FQHCs. Recommendations were derived from the various barriers and facilitators described by K.I.s. The use of the CFIR framework in this study to inform the data collection and analysis is consistent with other studies seeking to identify barriers and facilitators to implementing programs in primary care settings [34–36]. Furthermore, this study contributes to knowledge around the multilevel factors influencing the successful implementation of CRC screening programs in FQHCs [37]. Our findings outline several barriers to and facilitators of implementing a group-based *promotor*-led CRC screening program.

**Table 3:** Recommendations for implementing ML/MC CRC screening programs in FQHCs (organized by CFIR and non-CFIR domains)

CFIR Domains	Recommendations
**Intervention characteristics**	• Tailor education and outreach materials to fit the needs of the community
**Characteristics of the individual**	• Integrate *promotores* into the healthcare team as patient navigators and health educators to engage with Latino patients during the screening process.
**Inner setting**	
*Available resources*	• Utilize “inreach” and outreach teams to increase awareness and uptake of CRC screening • Offer free/discounted FIT kits for patients for in-person pick-up or mailed to their home • Utilize internal quality improvement teams to focus on specific patient care and metrics • Community-academic collaborations that can facilitate access to funding support for CRC programming
*Organizational incentives and rewards*	• Integrate friendly competition among care teams in the health center • Incorporate patient incentives such as raffles or monetary incentives may be an effective strategy for FIT kit or attendance at group-based events
*Relative priority*	• Prioritize screening and preventative care alongside treatment and other health education initiatives.
*Culture of the health center*	• Instill a culture of prevention among healthcare providers and staff at all levels.
*Goals & Feedback*	• Set internal goals within the health center and highlight the impact of efforts • Include a discussion of and progress toward HEDIS and health center quality measures during meetings with providers • Develop a multidisciplinary, internal task force to increase screening rates, develop incentive systems, and address workflow challenges
*Networks & Communication*	• Establish/enhance system-wide EHR notifications to indicate to providers which patients would benefit from group-based education and screening navigation.
**Outer Setting**	
*Cosmopolitanism*	• Build external relationships with social service and community agencies to address social determinants of health and to engage hard-to-reach populations.
*Patient Needs & Resources*	• Understand and mitigate patient barriers (i.e., transportation, time, health literacy) to screening.
*External Policy & Incentives*	• Consider national screening goals (i.e., UDS, HEDIS) to receive financial incentives for the health system. • Inform health centers about reimbursement models (i.e., federal block grants, insurance payment policies, and financial incentives), in which the organization benefits from implementing cost-effective CRC preventative services. • Consider creative funding models to allow for reimbursement (e.g., group-based with rotating 1:1 provider visits)
**Non-CFIR Domains**	
Existing infrastructure for CRC screening and health education	• Provide culturally responsive, bilingual CRC screening education materials

The FQHCs expressed interest in implementing a ML-MC CRC screening program, particularly if provided with pilot grant funding. As with many health education programs, implementing a CRC program is contingent on grant funding since reimbursement is largely not provided for health education activities [37]. This is a key takeaway that highlights the significance of community-research collaborations that can facilitate access to essential funding and resources. This collaboration is vital to employ research methodologies to optimize the intervention, allowing for tailoring of implementation strategies, feasibility assessments, and adaptation of the program to fit community and organizational needs. K.I.s in our study described FQHC leadership’s focus on national health system metrics, such as HEDIS and UDS measures, by which FQHCs are evaluated and can be renumerated if they meet stated goals. This is another important takeaway. Although Medicaid reimbursement for health education and promotor activities is limited, FQHC collaboration with academic research teams could allow for coordinated efforts to identify implementation strategies aimed at increasing CRC screening rates across the patient population. In doing so, FQHCs have an increased opportunity to meet national screening goals and potentially qualify for financial incentives for the health system. Therefore, when proposing new health education programs to FQHCs, it is essential to consider the external factors and incentives that could facilitate adoption and support from leadership.

As federal funding is essential to the operation of FQHCs, it is critical to identify strategies for health centers to access federal block grants, insurance payment policies, and financial incentives, in which the organization benefits from implementing cost-effective CRC preventative services. Physician performance evaluations and meeting HEDIS goals can serve as monetary incentives in FQHCs. Friendly competition among care teams was another strategy FQHCs found that motivated CRC preventative programming participation. Incorporating patient incentives such as raffles or monetary incentives may be an effective strategy for FIT kit completion among socioeconomically disadvantaged patients, which is also supported by recent literature [38]. 

As FQHCs face many of the same challenges as other health systems with limited staffing and large workloads, *promotor*-led programs can help lighten the load for health center staff. Given the limited capacity of providers, shifting the responsibility of preventive care and health education to *promotores* would allow the health center and administration to operate more efficiently. This is consistent with emerging research that highlights the value and effectiveness of using *promotores* as patient navigators and health educators to improve CRC screening rates in Latino populations [12, 24]. *Promotores* embedded within the healthcare team and engaged with patients in the CRC screening process, from initial patient outreach to completion of FIT kit tests, can improve access to and facilitation of preventative services [12]. *Promotores* reflect the target demographic, understand cultural nuances, and can facilitate language accessibility, improving access to healthcare for underserved populations that FQHCs are charged with reaching.

Several strategies could facilitate the implementation of a group-based promotor-led CRC screening program. At the policy level, national guidelines can continue encouraging FQHCs to increase CRC screening among their patient populations by setting ambitious screening targets and providing financial incentives to FQHCs that meet those goals. At the organizational level, FQHCs can prioritize and instill a culture of prevention among healthcare providers and staff at all levels so that screening and preventative care are valued alongside treatment. As described by one FQHC, friendly competition between health center sites increased CRC screening rates among patients. Promotions and incentive pay for providers can be linked with screening rates to encourage providers to refer patients to group-based education. Additionally, system-wide EHR notifications could indicate to providers which patients would benefit from group-based education and screening navigation. At the patient level, raffles or other financial incentives are effective in encouraging participation [38]. In addition, the group-based format of the CRC education sessions is attractive to patients who may feel more comfortable learning with a group of peers.

While there is much to be gained by FQHCs when implementing a group-based promotor-led cancer screening intervention, including meeting regulatory measures, financial incentives, and *promotores* to assist with patient education and navigation, some challenges remain. Access to diagnostic colonoscopies following a positive FIT test can be limited in underresourced communities. One of the FQHCs we interviewed serves a large patient population yet has only one gastroenterology specialist that will accept Medicaid patients for follow-up colonoscopies. Furthermore, if colorectal cancer were to be detected in a FQHC patient, access to cancer treatment would be even more challenging. Establishing strong community and academic partnerships will be essential for FQHCs to confront the challenges involved in CRC screening, diagnosis, and treatment.

The COVID-19 pandemic posed some challenges to the landscape of healthcare. Had these interviews taken place within the past three years, the long-standing issues of burnout, stress, insufficient staffing, limited funding, medical mistrust, and other factors, which were exacerbated during COVID-19, would likely have influenced K.I. perceptions of implementing a ML-MC CRC screening program at their respective FQHCs. Given the disruption of health education programming caused by COVID-19, K.I.s might have expressed concerns about the feasibility of delivering an in-person program or suggested alternative modalities, such as digital tools or telehealth. Although such strategies have effectively reduced numerous structural barriers to care for many communities, addressing the growing disparity in CRC screening among Latino communities necessitates culturally sensitive and appropriate approaches. COVID-19 has also underscored the importance of multifaceted, community-engaged programs in mitigating medical mistrust, promoting healthy behaviors, building resilience, and advancing health equity. Social connection and support from family and trusted members of the community are important cultural values held by Latino communities. ML-MC implementation strategies that leverage this form of resilience have the potential to reduce disparities related to CRC screening.

### Limitations and strengths

This study had several limitations that should be considered. First, although we attained the perspectives of FQHC leaders, providers, and staff, we did not interview other key stakeholders, such as medical assistants who interact directly with patients and may be involved in implementing CRC screening activities. Additionally, our sample did not include patients and potential recipients of the intervention. As such, findings may not reflect important patient needs and resources, as well as the various individual- and structural-level barriers to participation in a ML-MC CRC screening program. Furthermore, our sample size of FQHCs (*n* = 6) and K.I.s (*n* = 13) was small, limiting the generalizability of our findings. Findings also describe barriers and facilitators of a proposed group-based, promotor-led CRC screening intervention, and K.I.‘s responses were based on a summary sheet describing the JUNTOS intervention. However, data collected during the program’s actual implementation, both during and after, would likely elucidate additional barriers and facilitators to implementation. Lastly, it is worth noting that development of the interview guide took place prior to the publication of the updated CFIR [39]. Utilizing the updated CFIR might have led to more refined analyses, capturing more nuanced, defined constructs, thus enhancing precision and applicability. Future studies should consider collecting data from other program implementers, such as CHWs and K.I.s during program implementation. Despite the limitations, there are several strengths, including the input from stakeholders from a wide range of FQHCs. Furthermore, the current study is informed by a theoretical framework (i.e., CFIR).

Findings and recommendations from this research study can be used to inform academic-FQHC collaborations to implement ML-MC CRC screening interventions in FQHCs. Future studies should work collaboratively with FQHCs to design implementation strategies that leverage existing resources within FQHCs (e.g., outreach and “in-reach” initiatives, incentive and goal-setting systems, human and physical resources), while also expanding on external collaborations with community and social services organizations to engage hard-to-reach populations to increase CRC screening. Lastly, these strategies should prioritize national screening metrics (i.e., UDS, HEDIS) to enable FQHCs to capitalize on available financial incentives.

## Conclusion

Federally Qualified Health Centers play a vital role in delivering essential primary care to millions of underserved patients in the U.S. These centers have a unique opportunity to offer needed preventive care, such as screenings for CRC. Despite several motivations, including financial incentives, many FQHCs need help in meeting national metrics for CRC screening. Identifying and comprehending the barriers to implementation, along with developing effective strategies to overcome them, will be instrumental in addressing disparities in CRC screening practices and improving healthcare outcomes for diverse populations.

### Electronic supplementary material

Below is the link to the electronic supplementary material.

Supplementary Material 1

Supplementary Material 2

## Data Availability

The data that support the findings of this study are not publicly available due to protection of participants’ privacy and confidentiality and the small sample size and specificity of the study’s setting. However, data are available from the corresponding author upon reasonable request.

## Abbreviations

CRC: Colorectal cancer
FIT: Fecal immunochemical test
FQHC: Federally Qualified Health Center
MA: Medical Assistant
HEDIS: Healthcare Effectiveness Data and Information Set
UDS: Uniform Data System
IHP: Integrated Health Partners
K.I.: Key informant
ML-MC: Multi-level, Multicomponent
JUNTOS: Juntos Contra el Cáncer
