# Supplementary material for: Barriers and facilitators to implementing a multilevel, multicomponent intervention promoting colorectal cancer screening in health centers: a qualitative study of key informant perspectives
Source: BMC Health Serv Res. 2024 Mar 29;24:404. doi: 10.1186/s12913-024-10749-y (PMC10981354; doi:10.1186/s12913-024-10749-y)
Supplement: Supplementary file 1 — Supplementary Material 1 [file 12913_2024_10749_MOESM1_ESM.docx]

**Additional File 1**: Illustrative quotes from key informants discussing barriers and facilitators to implementing a ML-MC CRC screening program organized by CFIR domain

| **Barriers** | **Intervention characteristics** | **Facilitators** |
| --- | --- | --- |
| “I wouldn’t say it always has to be grant funded, but I think kind of as a pilot and a way to start, we would want to have that initial kind of pot of money.” | - Cost relating to intervention and implementation - Benefit of pre-established program - Potential for program to actively engage patients in their care | “I think anything that engages patients more on their care than, in terms of the reimbursement world will have benefits down the road as well if they’re coming back more and they’re now seeking health care when they would have just stayed home.” |
|  | **Characteristics of Individuals** |  |
| “I think that it may have been a good opportunity to teach a few more folks within, like bringing the clinic folks, right, the clinic administrators and some of the clinic team, maybe not the providers because they know it, but some of the nursing staff and other folks that were going to be encountering these folks that we’re sending your way, bringing them in and educating them on hey this what we’re doing, this is the intent.” | - Knowledge among clinic administrators and staff about the nature of the research and intervention - Such a program could help bridge the gaps in health education - Programs like this challenge cultural norms around the importance of CRC screening - Promotores who serve as peers and navigators can reduce barriers to screening | “As we’re transitioning from a higher level, from our volume to value, where we’re going from lots of visits to being paid for the quality of our care, and so that’s when positions like [promotores] start to come into play. And our mindset will definitely start to shift over the next few years to really focus on those types of positions that have a big impact, not necessarily on generation of visits but the impact on health education, getting them screening, our mindset will start to be shifting in that way anyways, so yes there’s a possibility for sustainability.”  “I feel like any kind of added resources to these types of efforts are welcomed. I think that we’ve learned when you have a focus on it…that just helps create that culture that these things are important and hopefully institutionalizes it more…” |
|  | **Inner Setting** |  |
|  | ***Available Resources*** |  |
| “We’re trying to work out a workflow where we can [implement a screening procedure] but it’s a lot of man hours.”  “Well, if you have one and a half MAs per clinician, then there’s always that pressure to room and to turn over the rooms and get the patient done, and there’s a time crunch. We have a lot of turnover for MAs.” | - Funding, space, staff time, and EMR systems - Staff turnover, competing priorities, and lack of leadership infrastructure - Internal teams focused on patient care metrics and outreach - Free or discounted FIT kits and colonoscopies - Patient navigators, *promotores*, and medical staff assisting with the CRC screening processes and follow-up that could facilitate implementation - EMRs that flag participants who are overdue for CRC screening and alert providers | “…we have had clinics that have created, that have hired navigators to really focus on encouraging the population what to do for colorectal cancer screening…”  “We do have a group of promotores that meet monthly and that’s a topic that they have been trained on and I think the American Cancer Society kind of came in and did some trainings so they can go out and educate. Their work and their impact isn’t tracked as well as it probably could be, but that is something that they enjoy hearing about and they confirm they want more of that information to share with their communities...”  “We are used to juggling space so that would be making sure you had the right resources on the day and then really plan ahead…not too much of an inconvenience.”  “[With the new electronic medical record system, we’ll have] a much higher level of point of care management, knowing each patient’s gaps, what we were saying earlier when we were identifying for what gap do you have for your normal course of your age and population, so we’re gonna have a lot more visibility…” |
|  |  |  |
|  | ***Organizational Incentives & Rewards*** |  |
| No barriers related to this code were reported. | - Goal-sharing awards, bonuses for providers, performance reviews, incentives, and promotions for meeting internal goals | “And then as a culture, they’re rewarded for that. They had a very robust reporting system for the average that the care teams broken down to the care teams, and then everything’s very, it’s broadcast to everybody, transparency, so that everybody knows how everyone is doing, and you know, clinicians are very competitive [laughter], so 'wait a minute, why are they doing better?' ... were able to reward those care teams for those efforts"  “We actually incentivized staff and patients for that because we wanted to bring in something that would make the educational component, we had these little—we kind of went through a short term communications campaign with our patients that wasn’t I think as relatable so we had some you know opportunity to learn from that but from an incentive the patients were excited about the incentive and I know that other community health centers have used incentives and I’ve seen their colorectal cancer screenings go way up…” |
|  | ***Relative Priority*** |  |
| “The challenge there is, you know, if you made an appointment, getting the care teams to think about the preventive stuff in the context of a bunch of other things.”  “There’s always higher value on one-on-one education, talking to a human being face-to-face...”  “I see the importance of the education and outreach component…but it would still kind of land rather low.” | - Value placed one-on-one interactions over group education - Higher priority to educate those diagnosed with chronic conditions than focusing on preventative care - Competing priorities from external policies to meet metrics for other diseases - Group education was a top priority | “Yes, [group education,] it’s top priority for our work plans for 2020.”  “We can make whatever needs to happen, happen. You know, if it seems valuable and it seems like it would benefit the patient, we can figure out how to make it work.” |
|  |  |  |
|  |  |  |
|  | ***Culture of the Clinic*** |  |
| "In our organization there's a lot of reservation when it comes to projects that are prevention projects." | - Absence and presence of values focused on prevention; Fostering a culture of prevention - Value placed on quality, integrity, and collaboration with community organizations and research institutions to focus on population health programs | “I think that we’ve learned when you have a focus on it… that just helps create that culture that these things are important and hopefully institutionalizes it more…we’ve talked a lot about creating a culture of prevention, both within our staff and for our patients, and so I think this would help us move the needle in that way.” |
|  | ***Goals & Feedback*** |  |
| “The past like three years it’s been a steady increase, and it’s not an easy needle to move.” | - Clinics not meeting goals for screening rates - Internal goals for increasing CRC screening rates that involve exceeding federal/state goals (HEDIS and UDS) - Internal teams/departments who discuss ways to increase screening, communicate feedback, and provide incentives to staff. | “…to convene task forces internally and we have a cancer screening task force led by a director of operations that’s multidisciplinary. There’s a lead clinician, there’s a variety of different folks from the clinic to really just brainstorm, like what are the efforts that we can implement to increase screening rates, what are the incentives that we can try, what are the different workflows that we should address? And that was the intent of that task force that’s still meeting and brainstorming.”  “used to try and look at all the different measures, the quality measures and try to improve our screening”  “being able to identify those positives that have been identified as a result of the screening and the impact that all this work has had in real life, not just in the numbers but to hear those stories…” |
|  | ***Networks & Communication*** |  |
| “Even though the *promotores* were employees of [FQHC]. They still kind of felt like outsiders in the clinic setting…I think if they were part of the team, there’s that relationship just builds that trust that continuity for the patients that are getting tested.” | - Need for *promotores* to integrate into the clinic workflow and build relationships with staff/providers - Provider and care team huddles for patient preparation and navigation in their workflow routines | “We don’t silo these departments because it doesn’t benefit the patients and we wanna prove broader audience and engagement in our overall outreach to the community and our internal community as well.”  “The patient retention engagement department also does a lot of patient outreach via phone for a variety of different reasons and they’re reaching out to different populations.” |
|  | **Outer Setting** |  |
|  | ***Cosmopolitanism*** |  |
| “Don't have a lot of partners because “it might just be our grants department…and then also time to set it up and build those relationships...plus location.”  “we haven’t really had any [partnerships with academics/grants] come to fruition though.” | - Presence or absence of partnerships with external organizations such as, IHP, clinics, school districts, churches, health plans or homeless centers, and university-based initiatives - Value building and maintaining external relationships with agencies and specialty clinics to address social determinants of health - Limited time to build relationships with external organizations | “And we’re part of the health center partners; all the large community health centers or FQHCs of San Diego, except for Family Health Centers, and we’ll sort of share our best practices with the rest of the groups there”  “We are exploring more ways… to engage the senior population more and like what other partners we could work with to do that… we do a little bit at the senior centers, and we have a good relationship with the city.” |
|  |  |  |
|  | ***Patient Needs & Resources*** |  |
| “It’s very difficult for people to come in for things that they’re not sick for, especially when they have to take work off”; It’s been hard for us to engage patients in preventive care.”  “Some of our patients do have that transportation challenge and so we feel that the mail-back would be a possibility to increase our return rate.”  “ … it was a lot of effort to get people to come to the workshop and then from the workshop to connect actually and get a test done and all of that stuff.” | - Transportation, time, and adherence to screening - Difficult to engage patients in preventative care - Patient desire for more group-based education - Value placed on in-person connection among Latino communities | "Thinking of Latino culture in particular we’re so much about that socialized and that connection of people… ‘pues el doctor me dijo que lo hiciera, lo voy hacer,’ right, but ‘yeah lo voy hacer’, but do I really do it? Right? Versus ‘oh a mi suegra le pasó esto, mas vale que te lo hagas.’ You know I think having those connections and relationships and familiarity just to build a community around the topic of the message helps create some of the deliverables we’re looking for” |
|  |  |  |
|  | ***External Policy & Incentives*** |  |
| “With the fee for service, it’s very difficult to be able to justify funding for prevention. So, until America becomes say a value-based system, it’s gonna be a challenge without grant funds and things to get it done” | - Lack of reimbursement for group sessions if the patient is not seen by a provider - Societal values surrounding health systems and health system change - Mandates from federal agencies (i.e., HEDIS or UDS measures) tied to financial implications are significant motivators for implementing CRC screening programs - Insurance companies incentivize CRC screening efforts to meet HEDIS measures - IHP negotiates contracts and provides funding to meet HEDIS measures (e.g., offset costs for FIT cards and testing) | “If we can keep our rate at 80% then we know we’re gonna get paid out at the end for the end of each year. That would more than pay for what we invested in”  “IHP... negotiates some of our insurance contracts for smaller nonprofit clinics in SD... help us with offsetting costs, so say buying FIT cards and sending them out for testing, they are able to look at aggregate rates and determine if a push is needed for outreach for that population based on how each clinic is performing”  "So that's a metric that all of our providers, it impacts our providers' performance evaluations annually. They have an opportunity for a bonus if they improve those rates. For clinic administration, it ties to their evaluation and performance as clinic administrators every year and what they're doing around that." |
|  |  |  |
