# Supplementary material for: Barriers and facilitators to implementing a multilevel, multicomponent intervention promoting colorectal cancer screening in health centers: a qualitative study of key informant perspectives
Source: BMC Health Serv Res. 2024 Mar 29;24:404. doi: 10.1186/s12913-024-10749-y (PMC10981354; doi:10.1186/s12913-024-10749-y)
Supplement: Supplementary file 2 — Supplementary Material 2 [file 12913_2024_10749_MOESM2_ESM.docx]

**Additional File 2:** Interview Guide for Healthcare Professionals (Clinical Personnel)

***Purpose:*** *The purpose of this interview is to learn and understand your clinic’s priorities, the decision-making processes, and programming related to colorectal cancer screening. Your answers to these questions will help us learn more about colorectal cancer care and provide guidance and structure for our proposed intervention and collaboration.*

***Disclaimer:*** *These questions were developed for a variety of clinic personnel; therefore you may feel that some questions do or do not apply to you. If that is the case, please let us know and try to answer to the best of your ability.*

**Section 1** *This first part will include questions about your clinic.*

1. **Please describe your role and/or job responsibilities at [Federally Qualified Health Center].**
2. **How would you describe the demographics of the adult patients that are seen at [FQHC]?** Probe: About how many patients does the clinic serve? Male or female? *[Inner Setting—Structural Characteristics]*
   1. About what percentage of these patients are Latinx/Hispanic?
3. **Does your clinic offer health education programs? If so, on what health topic(s) do they focus (e.g., cervical cancer, breast cancer, etc.)? How are they structured (e.g., workshops, one on one)? Who does the education (e.g., community health workers, *promotoras*, medical assistants, providers)?** Probe: To what extent does patient education take place inside/outside of the normal visit?
   1. *You mentioned X program.* How was this program started? Who were the individuals involved in moving this program forward and maintaining it?
4. **What factors does the clinic consider when deciding what health programs or initiatives to implement in your clinic?** Probes: strong demand, fits with values/goals, resources available, patient needs, reimbursement, other centers, cost. *[Intervention Characteristics—Evidence Strength & Quality]*
5. **To what degree does your FQHC interact or network with other academic institutions and community organizations?** *[Outer setting—Cosmopolitanism]*
   1. Have the partnerships been easy? What would you like to see in place? *[Outer setting-Cosmopolitanism]*
   2. If you have partnered with academic or community organizations, what value have these partnerships brought to your FQHC? Probe: resources, community visibility, etc*. [Inner Setting- Compatibility]*

*Now, I’m going to shift gears a little and ask you about programs that aim to increase colorectal cancer screening in your clinic.*

1. **How are patients notified when they are due or recommended for CRC screening?** Probe: methodology (only list if need more probing: verbal instructions by provider, mailed letter, phone call, etc.)
2. Who is involved in the process (e.g., providers, medical assistants, nurses, community health workers)
3. How often do providers or healthcare professionals talk with patients about colorectal cancer care and prevention? *[Available Resources]*
4. **What is the process that a patient goes through to get colorectal cancer screening using FOBT or FIT at your clinic?**
5. What is your clinic’s preferred protocol for CRC screening? (Colonoscopy, mailed FIT kits, mailed FOBT kits, etc.)
   1. What is the process of handing out the FIT or FOBT tests to patients? (Probe: is a MD prescription required vs. handing them out?).
6. For patients without insurance, what is the cost to the patient to obtain the FOBT or FIT?
   1. What is the cost for a colonoscopy?
7. **What approaches does your clinic use (or have used in the past) to increase the colorectal cancer screening among your patient population?** *[Inner Setting—Available Resources]*
   1. What are the main barriers you see among patients in accessing CRC screening? *[Outer Setting-Patient Needs and Resources]*
8. **What kind of goals or milestones is your FQHC trying to meet related to CRC screening?** *[Outer Setting—External Policies & Incentives]*

**Section 2 - Overall description of the project**

*The intervention we have developed, called JUNTOS Contra el Cáncer, seeks to increase CRC screening among Latinos 50 and over in San Diego and Imperial Counties. The intervention is implemented by promotores (community health workers) who recruit Latino men and women over 50 who are not up to date with CRC screening to attend one 2-hour workshop at the clinic or in a nearby community center (e.g., library). The workshop is didactic and interactive and includes information about colorectal cancer risk factors, screening tests, and reviews the instructions for completing a FIT test. Workshops are offered in both English and Spanish. Following the workshop, promotores conduct follow-up calls to help patients schedule provider visits at the clinic to get a FIT or colonoscopy ordered. Promotores then follow-up with patients to make sure they have attended the appointments and completed the screening test as ordered by their provider.*

1. **How interested or engaged would your staff be in supporting a new program like this?** *[Inner Setting—Implementation Climate—Tension for Change]*
2. **How receptive do you think patients would be in receiving information on the importance about CRC screening from a *promotor*/community health worker?** *[Characteristics of Individuals—Knowledge & Beliefs about the Intervention]*
3. **Given that our program would potentially recruit new patients to your FQHC, what barriers do you anticipate in engaging individuals who are not yet patients?** *[Inner Setting—Culture]*
4. **Compared to other programs and needs, where do you see this program fit within your FQHC? How high of a priority would a CRC screening intervention be for your FQHC?** *[Inner Setting—Implementation Climate—Relative Priority]*
5. **What kinds of indicators does your FQHC used to determine success? If we implemented our CRC screening program in your FQHC, how would you know that it is successful or not?** *[Process—Reflecting & Evaluating]*
6. **How would a program like this be sustained long-term? Is there any possibility of Medi-Cal reimbursement or health plan incentives for your clinic?** *[Outer Setting—External Policies & Incentives AND Intervention Characteristics--Cost]*
7. **Is there anything else that you would like to tell us about your experience working with adult patients or regarding CRC screening prevention at your FQHC?**
8. **What should [I/we] have asked you that [I/we] didn’t?**
